# Biosafety Analysis of Metabolites of *Streptomyces tauricus* Strain 19/97 M, Promising for the Production of Biological Products

**DOI:** 10.3390/bioengineering9030113

**Published:** 2022-03-11

**Authors:** Irina I. Gaidasheva, Tatiana L. Shashkova, Irina A. Orlovskaya, Tatyana I. Gromovykh

**Affiliations:** 1 Department of Biophysics, Siberian Federal University, 660041 Krasnoyarsk, Russia; 2 Department of Ecology and Environmental Management, Siberian Federal University, 660041 Krasnoyarsk, Russia; tatyana_eco@inbox.ru; 3 Laboratory of Stem Cell Immunobiology, Research Institute of Fundamental and Clinical Immunology, 630099 Novosibirsk, Russia; irorl@mail.ru; 4 Department of Biotechnology and Chemistry, Moscow Polytechnic University, 107023 Moscow, Russia; tigromovykh@rambler.ru

**Keywords:** biosafety, *Streptomyces tauricus*, against *Fusarium* fungi, metabolites, fungicidal biological preparation

## Abstract

A biosafety study was carried out concerning the metabolites of *Streptomyces tauricus* strain 19/97 M. This strain is a promising producer of biological preparations and shows antagonistic properties against *Fusarium* fungi, which cause Fusarium wilt disease. The strain has a pronounced biological activity against conifers, cereals and legumes. The treatment of planting material reduces infections, increases germination and furthers plant productivity. Using metabolites, we understood the culture liquid separated by filtration after the cultivation of the strain. Animals of different taxonomic affiliations were used as test objects: (CBA × C57BI/6) F1 hybrid mice (*Mus musculus)* (warm-blooded organisms), *Daphnia magna Straus* (planktonic crustaceans) and the unicellular alga *Chlorella vulgaris Beijer*. In the study, we were guided by the test standards for acute oral toxicity and irritation to the skin, mucous membranes of the eyes and inhalation toxicity. The research results showed that the metabolites of the strain are not acutely toxic to organisms of different taxonomic levels. The metabolites of the strain do not have an irritating effect on the skin and mucous membranes of warm-blooded animals. Based on the studies carried out, metabolites can be used for creating a fungicidal biological preparation.

## 1. Introduction

In soil microbocenoses, pathogenic micromycetes cause various plant diseases and produce mycotoxins that cause acute and chronic intoxication when ingested by animals and humans. Among them, Fusarium micromycetes are extremely aggressive. Scientific works provide evidence that fusariotoxins (FTs) contribute to the development of various diseases, provoke the growth of tumors and cause changes in plant metabolism, which leads to their death [1,2]. FTs have a multidirectional negative impact on all groups of organisms and are extremely dangerous. Therefore, the monitoring and development of methods for suppressing the growth and development of these pathogens are necessary. One of these methods of suppression is the creation of biopesticides based on strains of actinomycetes [3,4,5,6,7,8,9].

Research teams from different countries are involved in studying the antagonistic properties of actinomycetes concerning fungi and bacteria and developing preparations based on them. Research data have shown strong antagonistic properties of *Streptomyces avermitilis* against the agents of late blight and Alternaria [4]. The treatment of various plant cultures with a cell suspension of the *S. castelarensis* A4 strain resulted in a decrease in plant damage caused by leaf diseases, root rot, leaf and stem rust, Septoria blight and red-brown spot. Preparations of ActinovateR (*S. lydicus* WYEC 108), MycostopR (*S. griseoviridis* K61) and Micro108 (*S. lydicus* WYEC108) [5] were developed on the basis of actinomycetes. The metabolites synthesized by *Streptomyces* spp., including polyoxin D, streptomycin and kasugamycin, are also used to treat growing plants [6]. The antagonistic properties of actinomycetes against micromycetes of the genus *Fusarium*, *Alternaria*, *Puccinia*, yeast of the genus *Candida*, etc., have been proven [7]. 

As can be seen from the literature, a relatively large number of actinomycete strains deposited in industrial collections have been proposed for the production of biological preparations for plant protection. Still, the technology for their mass production is practically absent. 

In 1997, a research team isolated *Streptomyces tauricus* strain 19/97 M from the soil of a forest nursery in the Krasnoyarsk Territory. This strain was proposed as a potential producer of a biological preparation for stimulating the growth and protection of coniferous seedlings from pathogens caused by *Fusarium* and *Alternaria* fungi [10,11]. This strain is a promising producer for the creation of biological preparations based on its metabolites. To date, we have determined the optimal culture medium and optimal conditions for obtaining a plant protection biopesticide based on this microorganism. We calculated the kinematic characteristics of the cultivation process on different sources of carbon and nitrogen, and also selected a buffer system to maintain a constant pH of the process. The development of a biological preparation based on this strain is at the final stage. The preparation is very promising for use, as it showed good results for coniferous, legume and cereal crops, reduced the infection of seed material by 75% and increased seed germination by up to 40%. Forest and agronomic enterprises issued a positive test report.

Although we do not yet know the exact concentrations of substances that are part of the metabolites, we use them in their native form to stimulate plant growth based on the studies carried out. These substances are not concentrated, diluted or fragmented. It is in this form that we introduce the preparation into the soil and, therefore, study its toxicity. There is no need to concentrate this preparation according to the technology, and its dilution will not increase toxicity [11,12,13,14].

The use of antibiotics in agriculture contributes to an increase in the number of antibiotic-resistant microorganisms. Antibiotics should be used very carefully, given their possible penetration in food and the negative impact on human health. In agriculture, it is unacceptable to use medical antibiotics, such as tetracycline, streptomycin, penicillin, etc. Therefore, we carried out spectrophotometric, chromatographic and electrophoretic studies of the composition of the secondary metabolites of the strain, which showed the absence of tetracyclines, levomycytin and streptomycin in it [8].

During the cultivation of the strain, a change in the color of the culture liquid was also observed. Along with this, the antagonistic activity of the metabolites increased. The pigmented fraction had the greatest activity after separating the culture liquid into fractions. The pigment part accumulates primarily in the mycelium of the producer and can be isolated both from the native solution and the mycelium by extraction at acidic pH values. The UV spectra of the obtained compounds indicate that the isolated red antibiotics belong to quinones and anthracyclines and are similar in properties to rhodomycins and cinerubins [8].

The high interest in microbiological preparations of natural origin (biopesticides) existing in the world is accompanied by increased requirements for their effectiveness and safety, since they are directly introduced into the soil and actively interact with biota [1,2]. Before introducing preparations into the soil, we must investigate the environmental load on soil communities and toxicity to warm-blooded animals. In addition, we considered it crucial to investigate toxicity concerning aquatic microbiology, as we expect the preparation to enter water bodies and want to make sure that it is safe. There is not much information in the literature on the chemical composition and active component of this type of metabolites. It is mentioned that this species produces the antibiotic tavromycin [3]. This BSL-1 strain is assigned to work at genetic engineering facilities. Since the strain is considered safe, it can be assumed that the metabolites will also be non-toxic. However, according to the requirements for potential biological plant protection products, this must be practically confirmed. Therefore, the purpose of this study was to determine the biological safety of metabolites of *Streptomyces tauricus* strain 19/97 M concerning (CBA × C57BI/6) F1 hybrid mice [15,16,17], *Daphnia* (planktonic crustaceans) and the unicellular alga *Chlorella*, by methods for determining the acute toxicity by the reaction of aquatic organisms [18,19].

## 2. Materials and Methods

For the experiment, *Streptomyces tauricus* strain 19/97 M was cultivated on the optimal liquid medium with the following composition: starch—12.5 g/L, (NH_4_)_2_SO_4_—2 g/L, NaCl—1 g/L, K_2_HPO_4_—1 g/L, MgSO_4_·7H_2_O—1 g/L, buffer (K_2_HPO_4_/NaOH)—1 L. Phosphate-alkaline buffer (K_2_HPO_4_/NaOH) was used to neutralize acidic metabolic products.

The following process parameters were used for cultivation: cultivation temperature 27 °C, acidity of the medium pH 7.2, stirrer rotation speed 200 rpm, cultivation method—deep, duration of cultivation 120 h. The synthetic medium with the specified chemical composition does not possess antibiotic, antagonistic and stimulating properties [20,21,22,23]. 

On the fifth day, the biomass concentration is maximum and amounts to 2.57 ± 0.61 g/L. At the same time, maximum fungicidal activity is observed. The active metabolites of the strain diffuse into the medium, which is confirmed by the formation of a characteristic “red” color and an increase in the antagonistic and biological properties of the culture liquid with the metabolites dissolved in it, depending on the duration of cultivation [17]. To conduct a biosafety study, the cell mass was filtered. The solution after filtration with dissolved metabolites was used to determine the toxic effect on hybrid mice and aquatic organisms. 

**Figure 1:**
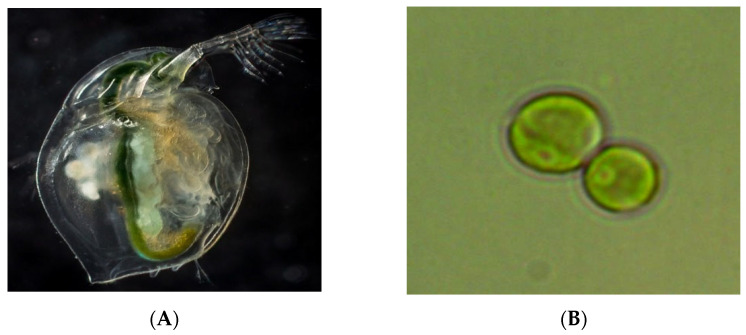
Test objects: (**A**) *Daphnia magna Straus*, (**B**) *Chlorella vulgaris Beijer*.

To assess the ecological safety of metabolites, freshwater cladoceran *Daphnia magna Straus* and the unicellular green alga *Chlorella vulgaris Beijer* were used as test organisms (Figure 1). 

The acute toxic effect on *Daphnia magna Straus* was assessed according to the bio testing method [5] on juveniles within 48 h of exposure in the tested samples. The exposure of Daphnia was carried out under the conditions of the R-2 climatostat (t = 20 ± 2 °C, photoperiod 12/12 h) in a rotating cassette of the УЭP-03 device for exposure of crustaceans. In the experiment, the filtrate was evaluated in its standard concentration and a number of its threefold dilutions. Cultivation water, which was optimal for the life of Daphnia, was used as a control and dilution water. The conclusion on the absence of toxic effect was made based on the calculated toxicity criterion, which does not go beyond the range of −30% to 20%, according to the method.

The toxic effect of the metabolite on the algae *Chlorella vulgaris Beijer* was evaluated by biotesting [19] by changing the optical density of the algae culture compared to the grown control variant. In the course of the study, common Beyer chlorella was cultivated on Tamiya nutrient medium (2%), which does not contain toxic substances, of the following composition: KNO_3_—2.5 g/L, MgSO_4_·7H_2_O—1.25 g/L, KH_2_PO_4_—0.156 g/L, FeC_6_H_5_O_7_—3 × 10^−3^ g/L, H_3_BO_3_—1.43 × 10^−3^ g/L, MnC_l2_·4H_2_O—0.90 × 10^−3^ g/L, ZnSO_4_·7H_2_O—0.11 × 10^−3^ g/L, MoO_3_—8.82 × 10^−6^ g/L, NH_4_VO_3_—11.48 × 10^−6^ g/L, distilled water.

The exposure was carried out in a KBM-05 multi-cell cultivator at the optimal mode (T = 36 ± 0.5 °C, average light intensity—60 W/m^2^) under the same and controlled conditions in terms of temperature, light intensity, CO_2_ supply and stirring for 22 h. In the experiment, the toxicity of the strain metabolites and a number of its threefold dilutions was evaluated. 

**Figure 2:**
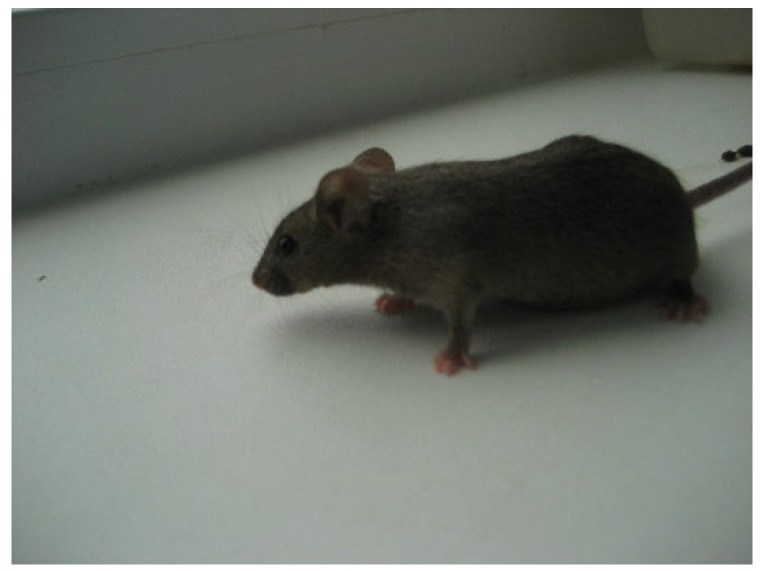
Clinically healthy (CBA × C57BI/6) F1 hybrid male mice.

Clinically healthy (CBA × C57BI/6) F1 hybrid male mice (*Mus musculus*) from the vivarium of Research Institute of Clinical Immunology, Siberian branch of the Russian Academy of Sciences, were used as warm-blooded animals. For scientific experiments, inbred mice with genetic homogeneity were used, ensuring the experimental results reproducibility. We observed the standard zoohygienic conditions for keeping animals (stocking density, airspeed, air humidity, water purity, bedding material, etc.) to obtain reliable data. For research, animals were kept in identical plastic cages, ten animals each, with free access to water (Figure 2). Feeding was carried out with natural food following the approved standards. The number of mice in the control and experimental groups was ten individuals, 8–10 weeks old, with a bodyweight of 23–25 g. All tests were carried out following the decision of the Local Ethical Committee, Research Institute of Fundamental and Clinical Immunology (Protocol No. 136).

Determination of acute oral toxicity, acute irritant/corrosive effect on the skin, irritant/corrosive effects on the eyes and acute inhalation toxicity was carried out in accordance with approved methods [15,16,17]. 

To assess the acute oral toxicity of *Streptomyces tauricus* 19/97 M, 200 mg/kg of filtrate was administered intragastrically to mice of the experimental group through gavage. Saline was administered to the mice of the control group in a similar way. The animals’ body weight was measured after 30 min, 2 h, 4 h, 24 h and every day for 14 days after administration. During the entire time, the number of dead animals was recorded. 

Before we tested *Streptomyces tauricus* 19/97 M for corrosive and irritating effect on the skin, we trimmed 1 × 1 cm areas on the back of the mice of both groups. Further, the trimmed skin area of the mice of the experimental group was covered with 0.5 mL of the filtrate. The same amount of saline solution was applied to the trimmed skin area of mice of the control group. The filtrate exposure was 4 h. The formation of erythema and edema was assessed after 1, 24, 48 and 72 h. The mice were also monitored for 14 days to exclude delayed irritation.

When studying the effect of metabolites on mucous membranes, one drop of the filtrate was dropped into the right eye of a mouse, and one drop of saline was dropped into the left eye. The formation of edema, redness, opacity of the cornea, iris and conjunctiva of the eye was assessed after 1, 24, 48 and 72 h. The mice were observed for 14 days to exclude delayed irritation.

To determine inhalation toxicity, 15 mL of the test solution was placed in a large desiccator and left under a lid for one day to fill the desiccator with volatile fractions of metabolites of *Streptomyces tauricus* strain 19/97 M. After 24 h, experimental mice were placed in a desiccator for one hour. According to the protocol, animals in the cage were observed for the appearance of changes in the skin and fur, eyes and mucous membranes, as well as the respiratory organs and autonomic and central nervous systems and, in addition, somatomotor activity and behavior patterns. We noted any differences between local and somatic effects, and paid attention to the occurrence of tremors, seizures, drooling, diarrhea, lethargy, drowsiness and coma. 

## 3. Results and Discussion

The popularity of pesticides derived from natural products has increased worldwide due to their high efficiency, environmental friendliness and favorable safety profile. A new direction in the use of actinomycetes as producers of antibiotic pesticides for plant protection deserves attention [24,25]. These pesticides, which include avermectins, spinosins, polynactin, tetramycin and their analogues, are already successfully used in plant protection in some countries. The development of scientific research on polyketide pesticides from actinomycetes reflects their importance for agriculture in recent decades [26]. However, studies have shown that these substances are toxic to most aquatic invertebrates and fish, so it is impossible to allow drugs based on them to enter reservoirs. The development of avermectin biotechnology has led to the constant improvement of the processes of searching for new producers of antibiotics synthesized by producers of this group and the need for their genetic modification. For example, strains of the species *Streptomyces avermitilis*, which are recommended for the production of avermectin biopesticides, are a source of genetic material for the construction of recombinant producers. Based on the industrial strain of *Streptomyces avermitilis*, which is a producer of anthelmintic macrocyclic lactones, avermectins, a universal model recombinant was designed for the heterologous expression of genes encoding the biosynthesis of secondary metabolites. However, in order to exclude the toxic effect of the secondary metabolites of actinomycetes, there is a need to search for less toxic metabolites of actinomycetes. Thus, moderately toxic metabolites of another potential producer, *S. castelarensis*, have already been identified for fish and bees.

The evaluation of the ecological safety of metabolites of the studied strain by biotesting methods on plant and animal organisms representing different trophic levels of freshwater ecosystems showed the absence of acute toxicity. Thus, the change in the optical density of the alga *Chlorella vulgaris Beijer* (Table 1) relative to the control variant of the experiment was within the standard values established by the toxicological analysis methods. The undiluted metabolite solution stimulated the growth of the alga, and with its three-fold and nine-fold dilution, the growth of the alga was lower than the control values, but these deviations were not statistically significant. 

A decrease in the survival rate of *Daphnia* by 50% or more in an acute toxicological experiment was not observed (Table 2); therefore, it was not possible to determine the LC50. Based on this, the metabolite solution can be considered as having no acute toxic effect. However, a decrease in survival rate of up to 60% was noted in a concentrated solution, as well as with a threefold dilution of the working solution. Based on this, a harmless dilution ratio (BKR10) was determined as equal to 20.5. 

**Figure 3:**
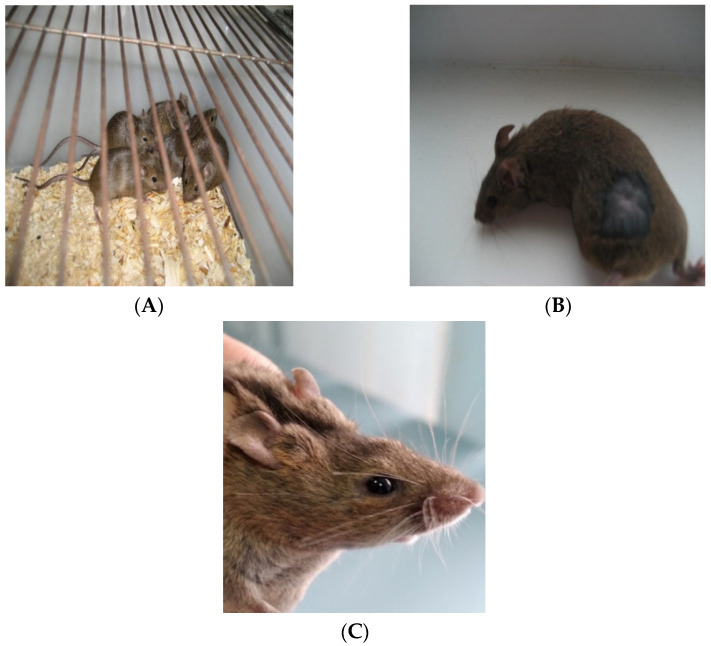
Mouse Hybrid F1 Line (CBA × C57BL/6) during the study: (**A**) reactions of the digestive system; (**B**) skin reactions; (**C**) effects on the mucous membrane of the eye.

The death of animals was not observed as the result of the study of acute toxicity and irritation. In addition, there were no changes in the general condition and behavior of the animals (Figure 3A) in the experimental groups compared to the animals in the control groups throughout the experiment. With the intragastric administration of the filtrate, changes in the bodyweight of mice were also not detected (Table 3 and Table 4). According to the statistical analysis, the results of the control values and the weight values of mice when using metabolites do not have significant differences.

When studying the irritating/corrosive effect on the skin of the metabolites of the strain in the mice of the experimental and control groups, no changes were noted on the examined skin area. Skin examination data are presented in Table 5.

The skin remained clean, without rash, redness, flaking and swelling throughout the study (Figure 3B). 

In an experiment to study the effect of the filtrate with dissolved metabolites of the strain on the mucous membranes of warm-blooded animals, the state of the experimental and control eyes was safe throughout the experiment. The test animals did not show any redness, increased tearing, discoloration of the cornea and iris or other discomforts (Figure 3B). Data on the state of the eyes of experimental animals are presented in Table 6.

When evaluating the effect of volatile fractions of metabolites on mice, it was shown that the behavior of the experimental group of mice did not differ from the control group. There were no visual changes in the skin and fur, eyes and mucous membranes, as well as in the respiratory system, autonomic and central nervous systems, and, in addition, in the somatomotor activity and behavior patterns. The mice of the experimental and control groups did not have tremors, convulsions, salivation, diarrhea, lethargy, drowsiness and coma.

## 4. Conclusions

An evaluation of the effect of metabolites of the *S. tauricus* strain 19/97 M studied by us did not show acute toxicity against freshwater organisms, which opens up the possibility of using this strain as an alternative to already known producers, and the need for its further study. Therefore, it is necessary to determine the chemical composition of its metabolites, and this is the subject of our further research. The positive forecasts on the prospects of using the studied strain are also increased by the tests conducted on warm-blooded animals—mice, which showed the absence of toxicity of the native solution of the culture fluid containing metabolites of the *S. tauricus* strain 19/97 M.

The study results of acute oral toxicity, acute inhalation toxicity and irritant/corrosive effects on the mucous membranes of the eyes and skin make it possible to classify the filtrate with the metabolites of this strain as substances that are hazardless and safe. We understand that, in the future, it is also necessary to conduct a visual and clinical examination of warm-blooded animals, including blood sampling and analysis and a post-mortem autopsy of mice to exclude the harmful effect of the preparation on the functioning of internal organs, as well as to exclude the formation of tumors and other negative consequences.

Thus, our assumption was confirmed by experimental data. Moreover, we assume that any dilution of metabolites and their transfer to an inert carrier will not lead to toxicity. 

Based on the presented results, we conclude that metabolites of *Streptomyces tauricus* strain 19/97 M can be used as the main active ingredient in creating a biological preparation with fungicidal and growth-stimulating action. The introduction of this drug into the soil will be safe for warm-blooded animals and humans.

## Figures and Tables

**Table 1:** Change in the optical density of the alga *Chlorella vulgaris Beijer* when exposed in the filtrate solution.

Optical Density	Control Group	Filtrate Dilution Ratio				
		1	3	9	27	81
	0.164 ± 0.006	0.207 ± 0.007	0.136 ± 0.015	0.152 ± 0.010	0.169 ± 0.005	0.166 ± 0.011
CT * (%)	0	−26.4	16.8	7.2	−3.4	−1.1

* CT (toxicity criterion)—the test sample has no toxic effect if CT is in the range of −30% to 20%.

**Table 2:** Survival of *Daphnia magna Straus* (%) according to the results of exposure in the filtrate solution.

	Control Group	Filtrate Dilution Ratio			
		1	3	9	27
Survival, %	100 ± 0	100 ± 0	97 ± 6	60 ± 30	100 ± 0

**Table 3:** Body weight of mice (g) with the introduction of the filtrate into the stomach (average values of 10 experimental individuals are indicated).

Control					
	Weight Before Introduction	After Introduction in an Hour			
		30 min	2	4	24
Average weight of 10 mice	24.1	24.1	24.1	24.2	24.7
Experiment					
	Weight before introduction	30 min	2	4	24
Average weight of 10 mice	24	24.3	24.3	24.4	24.9

**Table 4:** Body weight of mice (g) with the introduction of the filtrate into the stomach (average values of 10 experimental individuals are indicated).

Control														
		After Introduction in a Day												
	Weight Before Introduction	2	3	4	5	6	7	8	9	10	11	12	13	14
Average weight of 10 mice	24.1	24.6	24.9	24.8	24.9	25	25	24.9	25.2	25.2	24.7	24.6	24.9	24.9
Experiment														
	Weight before introduction	2	3	4	5	6	7	8	9	10	11	12	13	14
Average weight of 10 mice	24.3	24.8	25.1	25	25.1	25.2	25.2	25.1	25.4	25.4	24.9	24.8	25.1	25

**Table 5:** Skin condition of experimental animals when applying the filtrate.

Skin Condition	Number of Mice in			
	1 h	24 h	48 h	72 h
Erythema formation				
None	10	10	10	10
Edema formation				
None	10	10	10	10

**Table 6:** Skin condition of experimental animals.

Eye Condition	Number of Mice in			
	1 h	24 h	48 h	72 h
Cornea				
No ulceration or opacity	10	10	10	10
Iris				
Normal	10	10	10	10
Conjunctiva				
No redness	10	10	10	10

## Data Availability

The data presented in this study are available on request from the corresponding author.
